# New Monoclonal Antibodies against a Novel Subtype of Shiga Toxin 1 Produced by *Enterobacter cloacae* and Their Use in Analysis of Human Serum

**DOI:** 10.1128/mSphere.00099-15

**Published:** 2016-02-17

**Authors:** Craig Skinner, Stephanie Patfield, Rowaida Khalil, Qiulian Kong, Xiaohua He

**Affiliations:** Western Regional Research Center, U.S. Department of Agriculture, Agricultural Research Service, Albany, California, USA; University of Rochester

**Keywords:** *Enterobacter*, Shiga toxins, Stx1e, immunoassays, monoclonal antibodies

## Abstract

Shiga toxin (Stx) is a major virulence factor of several bacterial pathogens that cause potentially fatal illness, including *Escherichia coli* and *Shigella* spp*.* The continual emergence of new subtypes of Stxs presents challenges for the clinical diagnosis of infections caused by Stx-producing organisms. Here, we report the development of four new monoclonal antibodies (MAbs) against Stx1e, a novel subtype of Stx1 that was produced by an *Enterobacter cloacae* strain and had limited reactivity with existing anti-Stx1 antibodies. Western blot analysis indicates that these MAbs were Stx1 specific, bound to the A subunit, and had distinct preferences for subtypes of Stx1. Of the four MAbs, Stx1e-2 was capable of partially neutralizing cytotoxicities derived from Stx1e in Vero cells. Enzyme-linked immunosorbent assays assembled with these high-affinity MAbs detected Stx1e at concentrations as low as 4.8 pg/ml in phosphate-buffered saline and 53.6 pg/ml in spiked human serum samples and were also capable of distinguishing Stx1e-producing strains in enriched cultures. These assays may therefore have clinical value in diagnosing Stx1e-producing bacterial infection. Additionally, characteristics of Stx1e, such as the origin of *stx1e* genes, conditions for toxin expression, receptor binding, and cytotoxicity, were investigated with the new antibodies developed in this study. This information should be useful for further understanding the clinical significance and prevalence of Stx1e-harboring *E. cloacae* and other organisms.

**IMPORTANCE** Stxs are among the most clinically important virulence factors of *Shigella* and enterohemorrhagic *Escherichia coli*. There are many varieties of Stx, and although Stx1a and Stx2a are the most common and widely distributed types of Stx, new variants of Stx are continually emerging. These new variants of Stx can be challenging to detect, since most Stx detection kits are optimized for the detection of Stx1a and Stx2a. Stx1e, recently discovered in an atypical host (*Enterobacter cloacae*), is undetectable by many Stx assays. To formulate new assays for the detection of Stx1e, we generated four new MAbs that recognize this Stx subtype. Using these antibodies, we generated an assay capable of detecting Stx1e at low picogram-per-milliliter concentrations. This assay is also compatible with a human serum matrix, suggesting that it may have utility for the clinical detection and diagnosis of Stx1e-associated infections.

## Abstract (precis)

Stxs are among the most clinically important virulence factors of *Shigella* and enterohemorrhagic *Escherichia coli*. There are many varieties of Stx, and although Stx1a and Stx2a are the most common and widely distributed types of Stx, new variants of Stx are continually emerging. These new variants of Stx can be challenging to detect, since most Stx detection kits are optimized for the detection of Stx1a and Stx2a. Stx1e, recently discovered in an atypical host (*Enterobacter cloacae*), is undetectable by many Stx assays. To formulate new assays for the detection of Stx1e, we generated four new MAbs that recognize this Stx subtype. Using these antibodies, we generated an assay capable of detecting Stx1e at low picogram-per-milliliter concentrations. This assay is also compatible with a human serum matrix, suggesting that it may have utility for the clinical detection and diagnosis of Stx1e-associated infections.

## INTRODUCTION

Shiga toxin (Stx)-producing *Escherichia coli* (STEC) is a worldwide health concern affecting an estimated 265,000 United States citizens and about 3 million persons globally each year (1, 2). However, STEC is a harmless component of the natural flora of many ruminants (1, 3) and is therefore nearly ubiquitous in the environment. STEC infection has a variety of clinical outcomes, ranging from mild diarrhea to hemorrhagic colitis (bloody diarrhea) and potentially deadly hemolytic-uremic syndrome (HUS) (4). Stx is among the most clinically relevant virulence factors of STEC and plays a critical role in the development of hemorrhagic colitis and HUS (4, 5). Two dissimilar types of Stx have evolved in *E. coli*, Stx1 (of which there are three subtypes, Stx1a, Stx1c, and Stx1d) and Stx2 (including seven subtypes, Stx2a through Stx2g) (6). All Stxs have similar macromolecular structures consisting of a single catalytic A subunit and five receptor-binding B subunits (an AB_5_ configuration), but variations in immunological reactivity, receptor preference, and toxin potency were found among subtypes of Stxs (7–9) that present challenges for the detection of these toxins and evaluation of their prevalence, distribution, and clinical relevance.

The genes encoding Stxs are found mainly in the *Escherichia* and *Shigella* genera. They are usually carried by functional lambdoid phages (in the case of Stx2 and some Stx1 forms in *E. coli*) or now-defunct lambdoid remnants (in the case of *Shigella dysenteriae* Stx) (10). Because of the ability of lambdoid phages to infect other bacterial hosts within the family *Enterobacteriaceae*, *stx* genes have been discovered in a variety of bacteria, including *Citrobacter freundii* (11, 12), *Enterobacter cloacae* (13), *Acinetobacter haemolyticus* (14), *Aeromonas* sp*.* (15), and even the distantly related genus *Enterococcus* (16). However, the presence of *stx* genes in these atypical hosts waned after repeated subcultures, suggesting that the phages may not propagate efficiently within them or that the *stx* genes themselves are unstable.

Recently, the California Department of Public Health (Richmond, CA) identified an Stx1-producing *E. cloacae* strain, M12X01451, from a human clinical specimen (17). The case patient had nonbloody diarrhea and abdominal cramping that persisted for 5 days. Unlike the other atypical Stx hosts, *E. cloacae* strain M12X01451 was shown to be a stable carrier of an *stx1* gene. The M12X01451 *stx1* gene showed 87% amino acid sequence identity to *stx1c*, and the gene product was named Stx1e on the basis of sequence dissimilarity with known Stx1 subtypes and the recent nomenclature recommendations (17, 18). This Stx1e is the first new subtype of Stx1 to be discovered in an organism other than *Shigella* or *E. coli* (17). It was found to be toxic to Vero cells but was not neutralized by the commonly used 13C4 anti-Stx1 monoclonal antibodies (MAbs) and was recognized poorly by commercial Stx1 detection kits (17). This suggests that existing Stx1-specific antibodies may be of limited use in Stx1e analysis and that new antibodies and better diagnostic tools are needed for Stx1e. Here, we describe the generation of high-affinity MAbs that recognize Stx1e and their use for characterizing and detecting this toxin in human serum samples.

## RESULTS

### Generation and characterization of MAbs against Stx1e.

**FIG 1:**
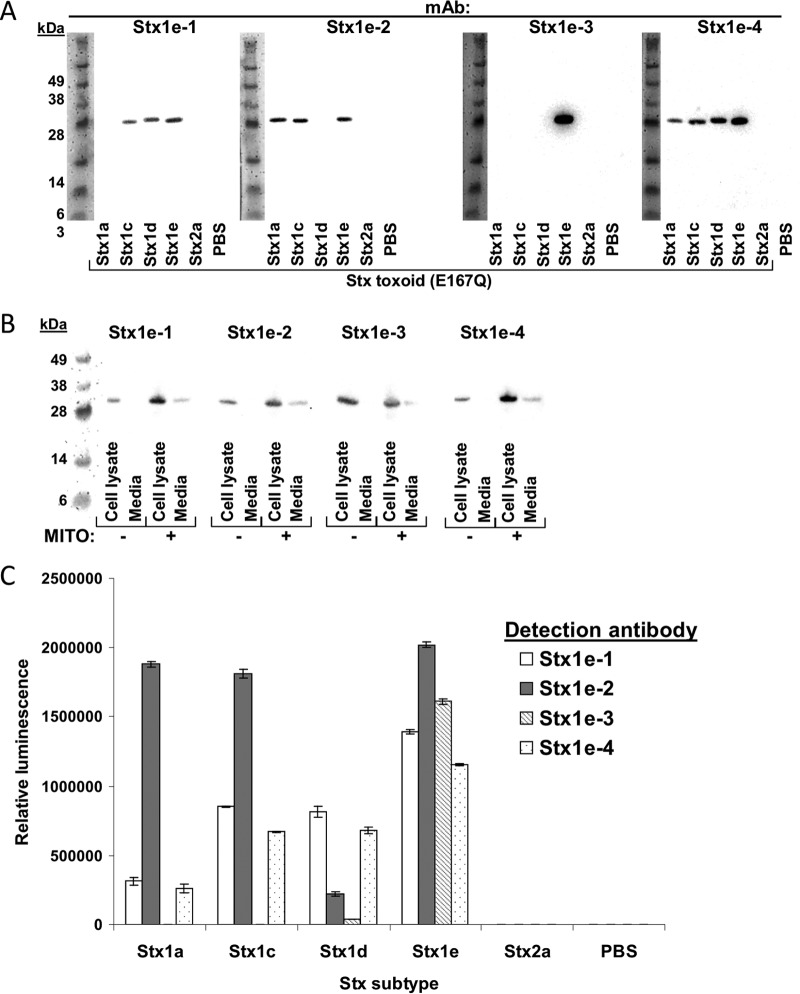
Stx1e antibody subtype and subunit specificity. (A) Western immunoblot assays with anti-Stx1e MAbs and purified toxoid (50 ng/lane). Stx1e antibodies were used at 1 µg/ml. (B) Western blot assays with anti-Stx1e MAbs on lysate or medium samples from *E. cloacae* MX1201451 induced with mitomycin C (MITO; final concentration, 50 ng/ml) or not induced, as indicated. (C) Direct ELISA of the toxoids indicated with 1 µg/ml toxoid and 1 µg/ml MAb or PAb.

Recombinant catalytically inactive Stx1e (E167Q) toxoid was expressed in *E. coli* and purified by multiple-step chromatography (including anion exchange, hydrophobic interaction, and gel filtration). The final product (see Fig. S1 in the supplemental material) was injected into mice. By standard hybridoma fusion techniques, splenocytes from immunized mice were fused to mouse myeloma cells (SP2/0). A total of 2,880 wells containing 10 to 100 hybridomas per well were screened for the ability to bind Stx1e toxoid. After three to five rounds of clonal selection and recovery, four hybridomas that produce high-affinity anti-Stx1e MAbs were selected for further characterization. The isotypes, dissociation constants, and specificities of the antibodies produced by these four hybridoma cell lines are summarized in Table 1. All four antibodies were found to bind the A subunit of Stx1e toxoid in Western immunoblot assays (Fig. 1A). In addition to Stx1e, MAb Stx1e-1 also recognized Stx1c and Stx1d but not Stx1a toxoid. MAb Stx1e-2 detected Stx1a, Stx1c, and Stx1e but not Stx1d. MAb Stx1e-4 recognized all four subtypes of Stx1, although it detected Stx1a poorly. MAb Stx1e-3 had a unique specificity for only Stx1e. None of these MAbs cross-reacted with Stx2a. As an essential confirmation, these antibodies were tested by Western immunoblot assay for the ability to detect Stx1e produced by bacterial strain M12X01451. All four antibodies did recognize Stx1e in both cell lysate and cell-free medium, and as with many *E. coli* strains that express Stx1 or Stx2, mitomycin C greatly increased the amount of Stx1e produced by *E. cloacae* M12X01451 (Fig. 1B).

10.1128/mSphere.00099-15.1 Figure S1  Purity of the Stx1e (E167Q) toxoid used for mouse injection and the Stx1e toxin used for cytotoxicity experiments, as illustrated by SDS-PAGE and Coomassie blue staining. Toxin/toxoid was run at 50 ng/well, and pure Stx1a was included for comparison. Download Figure S1, PPTX file, 0.3 MB. Copyright © 2016 Skinner et al. 2016 Skinner et al. This content is distributed under the terms of the Creative Commons Attribution 4.0 International license.

**TABLE 1:** Characteristics of the antibodies used in this study

Antibody	Isotype	Avg *K_D_* (10^−9^ M) ± SD	Subunit	Specificity^a^
Stx1e-1	IgG1, kappa	5.2 ± 2.2	A	Stx1c, -d, -e
Stx1e-2	IgG2a, kappa	1.0 ± 0.2	A	Stx1a, -c, -e
Stx1e-3	IgG1, kappa	2.6 ± 1.4	A	Stx1e
Stx1e-4	IgG1, kappa	NA	A	Stx1a, -c, -d, -e

a Based on Western immunoblotting.

**FIG 2:**
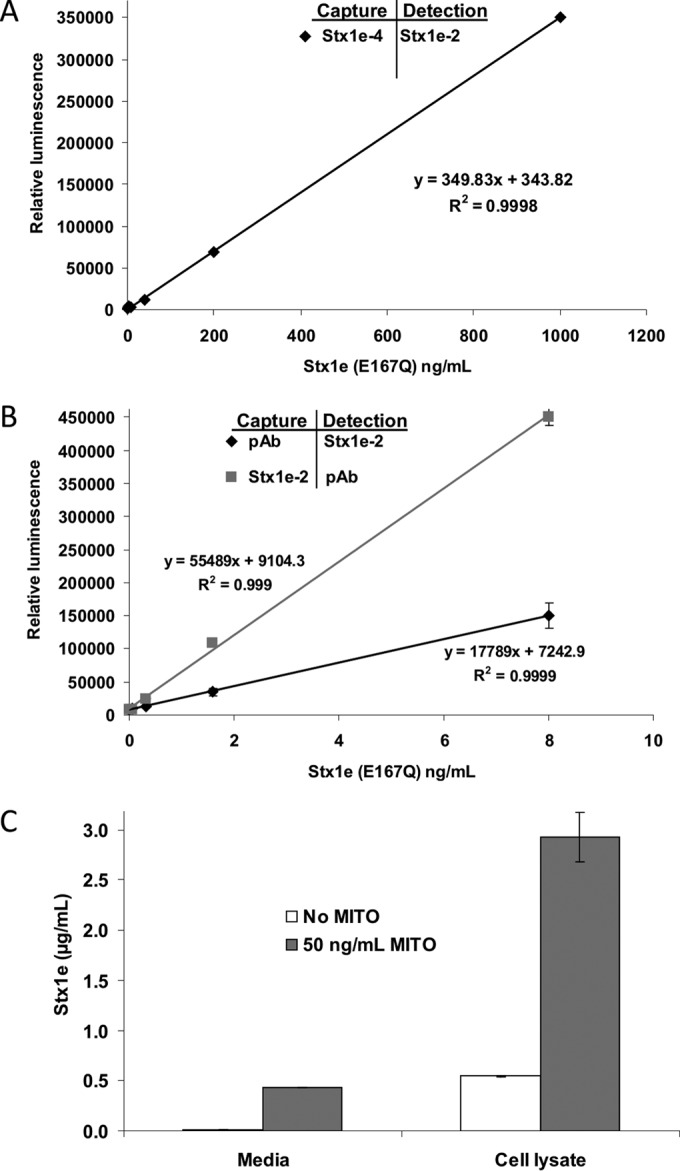
ELISAs for Stx1e detection. (A) Detection of Stx1e by MAb sandwich ELISA. Capture MAbs were used at 5 µg/ml; detection MAbs were used at 1 µg/ml. (B) Detection of Stx1e by MAb Stx1e-2/PAb or PAb/Stx1e-2 sandwich ELISA. The capture antibody was used at 5 µg/ml; the detection antibody was used at 1 µg/ml. (C) Detection of Stx1e in M12X01451 medium and cell lysate with the PAb/Stx1e-2 antibody combination in sandwich ELISA. MITO, mitomycin C.

The subtype specificity of these four MAbs was also tested by direct enzyme-linked immunosorbent assays (ELISAs), and the results differed slightly from those of the Western immunoblot assays (this may be due to sensitivity differences or epitope linearity). Stx1e-1 and Stx1e-2 recognized all four subtypes of Stx1 (to various degrees) in direct ELISAs (Fig. 2C). Similar to the Western blot assays, none of the MAbs cross-reacted with Stx2a in direct ELISAs.

### Assays for Stx1e detection.

To select antibodies that could be used for sensitive detection of Stx1e in immunoassays, four MAbs plus one previously developed polyclonal antibody (PAb) against Stx1a (see Fig. S2 in the supplemental material) were paired in all possible combinations and evaluated by sandwich ELISAs (see Fig. S3 in the supplemental material). Sandwich, or indirect, ELISAs are often more sensitive when applied to complex matrices because of the lack of competition for plate binding. Antibody combinations PAb/Stx1e-2, Stx1e-2/PAb, and Stx1e-4/Stx1e-2 were superior to the others and were therefore further evaluated for the ability to recognize Stx1a, Stx1c, and Stx1d. The sandwich ELISA with MAbs Stx1e-4/Stx1e-2 detected Stx1a and Stx1c very poorly but cross-reacted with Stx1d (see Fig. S4 in the supplemental material). The limit of detection for Stx1e with this ELISA was 2.3 ng/ml (Fig. 2A). The PAb/Stx1e-2 and Stx1e-2/PAb combinations were more sensitive and less specific, recognizing all four Stx1 subtypes (see Fig. S4 in the supplemental material) with limits of detection (LODs) for Stx1e of 4.3 and 7.8 pg/ml, respectively (Fig. 2B).

10.1128/mSphere.00099-15.2 Figure S2  (A) Stx1 subtype specificities of the anti-Stx1 PAb, evaluated by direct ELISA, with pure toxoid at 1 µg/ml in PBS. (B) Stx1e recognition of the anti-Stx1 PAb. Samples include concentrated (5×) filtered medium of the strains indicated and 50 ng of purified Stx1e toxin. Goat anti-rabbit alkaline phosphatase was used to develop the Western blot assay. Download Figure S2, PPTX file, 0.9 MB. Copyright © 2016 Skinner et al. 2016 Skinner et al. This content is distributed under the terms of the Creative Commons Attribution 4.0 International license.

10.1128/mSphere.00099-15.3 Figure S3  Antibody combinations for sandwich ELISAs. Antibodies used for capture were paired with detection antibodies in all possible combinations (except PAb/PAb). ELISAs were conducted with 1 µg/ml Stx1e (E167Q) toxoid. Download Figure S3, PPTX file, 0.1 MB. Copyright © 2016 Skinner et al. 2016 Skinner et al. This content is distributed under the terms of the Creative Commons Attribution 4.0 International license.

10.1128/mSphere.00099-15.4 Figure S4  Specificities of the most sensitive Stx1e ELISAs. (A) MAb combination ELISAs (Stx1e-1/Stx1e-2 and Stx1e-4/Stx1e-2) were used to detect the four subtypes of Stx1. (B) The most sensitive Stx1e assays (PAb/Stx1e-2 and Stx1e-2/PAb) were tested for their specificity for all four subtypes of Stx1. Download Figure S4, PPTX file, 0.1 MB. Copyright © 2016 Skinner et al. 2016 Skinner et al. This content is distributed under the terms of the Creative Commons Attribution 4.0 International license.

Using purified Stx1e (E167Q) toxoid as a standard and a sandwich ELISA with the Stx1e-2/PAb antibody combination, we were able to estimate the amount of Stx1e present in M12X01451 cell-free culture medium. Stx1e was present at 7.7 ng/ml without mitomycin C induction and at 432.3 ng/ml with mitomycin C, an induction of 56.3-fold (Fig. 2C). However, the quantity of cell-free Stx1e was dwarfed by the amount remaining in the cells. Without mitomycin C induction, the cell lysate contained 545.8 ng/ml, and with induction, it contained 2.93 µg/ml, an increase of 5.4-fold. Such a dramatic induction upon mitomycin C treatment further reinforces the hypothesis that Stx1e expression is controlled by a phage promoter.

### Stx1e is phage encoded, and the phage is active.

**FIG 3:**
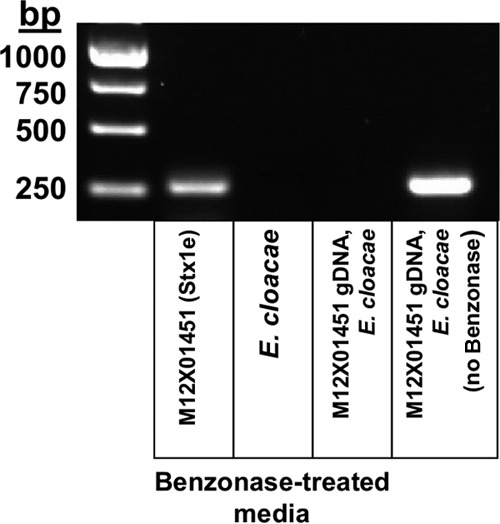
Protection of the *stx1e* gene in filtered medium. A PCR of the samples indicated was performed with or without Benzonase treatment (to eliminate unprotected DNA), as indicated.

Stx2 is encoded by an active lambdoid phage, and this enables its transfer among other pathogenic and nonpathogenic strains of *E. coli* (19). The Stx of *Shigella* spp*.* is also associated with phage sequences, but the phage is defective and can no longer undergo the lytic phase of its life cycle (10). Low concentrations of iron induce the expression of Stx in *Shigella* spp*.* (20). Some Stx1-containing phages are thought to be defective as well and are likewise inducible by low iron concentrations (21). To test whether the *stx1e* gene is part of the bacterial genomic DNA (gDNA), carried by a latent phage, or carried by an active phage, we determined whether we can detect the *stx1e* gene in filter-sterilized bacterial medium. An *stx1e*-specific PCR was developed by using regions within the *stx1e* A subunit DNA that are distinct among Stx1 subtypes (see Table S1 in the supplemental material). Samples including filter-sterilized medium from Stx1e-expressing strain M12X01451 or the *E. cloacae* type strain (ATCC 13047) were treated with Benzonase prior to PCR to ensure that the medium contained no bacterial gDNA (or mRNA). Of the media of these three strains, only that of Stx1e-expressing M12X01451 showed an *stx1e*-positive band (Fig. 3). A control PCR of the genomic control gene *wrbA* yielded no band for the medium-containing Benzonase-treated samples (see Fig. S5 in the supplemental material), which indicates that the filtered medium did not contain any intact bacterial cells. gDNA was absent as well: no PCR product was detected in samples containing M12X01451 gDNA pretreated with Benzonase. These results suggest that the *stx1e* gene is carried by an active phage.

10.1128/mSphere.00099-15.5 Figure S5  Control PCR for bacterial contamination of the samples in Fig. 3. A PCR encompassing the *wrbA* gene was conducted for the samples above. Download Figure S5, PPTX file, 0.6 MB. Copyright © 2016 Skinner et al. 2016 Skinner et al. This content is distributed under the terms of the Creative Commons Attribution 4.0 International license.

10.1128/mSphere.00099-15.8 Table S1  Primers used in this study. Download Table S1, PPTX file, 0.1 MB. Copyright © 2016 Skinner et al. 2016 Skinner et al. This content is distributed under the terms of the Creative Commons Attribution 4.0 International license.

### Receptor preference and antibody neutralization of Stx1e cytotoxicity.

**FIG 4:**
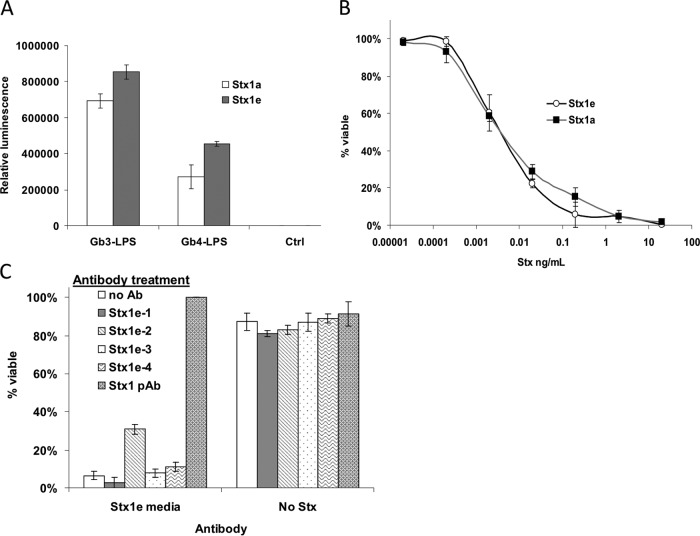
Receptor binding and neutralization of Stx1e. Receptor preference for Stx1e was analyzed by using *E. coli* cells that display Gb3-LPS or Gb4-LPS on their surface. (A) Gb3 and Gb4 binding of Stx1e toxoid, compared to Stx1a toxoid. Strains expressing LPS, Gb3-LPS, and Gb4-LPS were used in this assay, as well as MAb Stx1e-2 (at 1 µg/ml). Ctrl, control. (B) Stx1e cytotoxicity as measured by Vero cell assay. Cells were seeded at 10^4^/well. Stx1e was purified from M12X01451 lysate. Pure Stx1a was used as a control. (C) Neutralization of Stx1e cytotoxicity for Vero cells. An M12X01451 overnight culture containing Stx1e and induced by mitomycin C was filtered and added (0.1 µl/well) to Vero cell medium containing 10 µg/ml antibody and incubated for 1 h. This mixture was then added to wells (seeded with 10^4^ cells/well) and incubated for 24 h.

Stx1 recognizes the globotriose saccharide on the surface of target cells, but unlike Stx2, the lipid anchor for the sugar does not affect its binding efficiency (22). Stx1a is therefore capable of binding both Gb3 and Gb4 (22). To test whether Stx1e has a similar receptor binding preference, we utilized MAbs developed in this study and bacterial cells that express Gb3-lipopolysaccharide (LPS) or Gb4-LPS upon their surface or an empty vector as a control (23, 24). Stx1e (E167Q) bound to both Gb3- and Gb4-LPS, similar to the Stx1a (E167Q) control, which also bound to both sugars (Fig. 4A).

To determine the cytotoxicity of the Stx1e subtype, we treated Vero (green monkey kidney) cells with various concentrations of purified Stx1e (from strain M12X01451). The 50% cytotoxic dose (CD_50_) of Stx1e was determined to be 3.7 pg/ml, almost identical to the CD_50_ of Stx1a (4.2 pg/ml) (Fig. 4B; see Fig. S6 in the supplemental material). To examine the ability of our anti-Stx1e antibodies to neutralize native Stx1e produced by strain M12X01451, Stx1e-containing bacterial supernatants were preincubated with antibodies before Vero cell treatment. Although most of the MAbs did not affect the toxicity of Stx1e-containing cell-free medium, 31% of the toxin was neutralized by Stx1e-2 and the Stx1 polyclonal fully neutralized Stx1e toxicity (Fig. 4C) at 10 µg/ml antibody. This is an unsurprising result since A subunit-specific MAbs are frequently less effective at neutralizing Stx in cell culture than are B subunit-specific MAbs or PAbs. The ability of the Stx1 PAb to fully detoxify the M12X01451 culture supernatant suggests that no other toxins were present and that Stx1e was primarily responsible for toxicity to Vero cells.

10.1128/mSphere.00099-15.6 Figure S6  Photographs of Vero cells at the conclusion of the cytotoxicity assay. These photos correspond to the appropriate sample wells in Fig. 4. Download Figure S6, PPTX file, 3.4 MB. Copyright © 2016 Skinner et al. 2016 Skinner et al. This content is distributed under the terms of the Creative Commons Attribution 4.0 International license.

### Detection of Stx1e in human serum.

**FIG 5:**
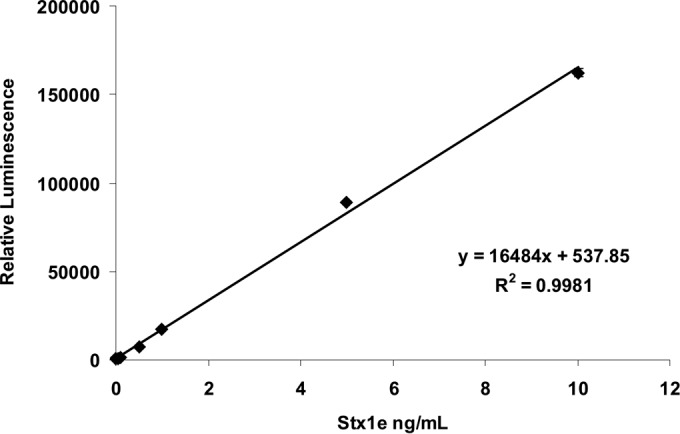
Detection of Stx1e in human serum. Purified Stx1e in serial dilutions was used to spike human serum (diluted 10-fold in PBS) and analyzed by ELISA. Detection and HRP-conjugated antibodies were preincubated with human serum prior to use in this assay.

Stxs are frequently associated with the complication of infections caused by Stx-producing organisms. The occurrence of bacterial strains expressing new subtypes of Stxs presents challenges for monitoring the level of serum Stxs and their relationship to the clinical course of bacterial infection toward HUS development. Since strain M12X01451 was originally isolated from a human patient (17), there could be diagnostic applications for an assay that detects Stx1e in human serum. Therefore, we evaluated whether one of our most sensitive assays (Stx1e-2 as a capture antibody, the Stx1 PAb for detection) is compatible with the human serum matrix. Purified Stx1e was used to spike pooled human serum diluted 10-fold in phosphate-buffered saline (PBS). It was found that this Stx1e ELISA had considerable background noise (data not shown). However, when the detection PAb and goat anti-rabbit horseradish peroxidase (HRP)-conjugated antibody were preincubated with human serum for 1 h at 25°C, the background became negligible (Fig. 5). The final LOD with this assay was 53.6 pg/ml.

## DISCUSSION

As the first Stx to propagate stably within the clinically relevant organism *E. cloacae*, Stx1e is a prototype for non-*E. coli* (or *Shigella*) Stxs. Here, we report the development of four A subunit-specific MAbs that recognize Stx1e. Using these new molecular tools, we developed sensitive assays capable of detecting Stx1e at low picogram-per-milliliter concentrations. Utilizing these assays, we determined that mitomycin C induces Stx1e expression in *E. cloacae* M12X01451 and that most of the Stx1e in both induced and noninduced cultures is cell associated. These assays could have clinical diagnostic applications as well, since they are compatible with human serum. We additionally demonstrated affinity of Stx1e to its presumed receptor Gb3 (and Gb4) and measured its cytotoxicity to Vero cells. Finally, we provided evidence that the *stx1e* operon resides upon a mobile element, an active phage, suggesting that the *stx1e* genes could be common and providing information on the origin of this Stx1 subtype.

Although it is not thought to be a major cause of gastrointestinal illness, *E. cloacae* could be a larger contributor to food contamination than suspected. Many rapid diagnostic tests for foodborne pathogens focus on the common culprits (norovirus, *Salmonella*, *Listeria*, *E. coli*, and *Campylobacter*), and standard microbiological tools that detect and/or differentiate *E. coli* are often not analyzed for *E. cloacae* (MacConkey agar plates, for example). It is possible that intestinal *E. cloacae* infections are generally less severe than STEC infections and therefore may not result in hospital admission and go unreported. The presence of a phage-carried Stx in some strains of pathogenic *E. cloacae* could change the frequency of hospital admissions caused by *E. cloacae* gastrointestinal pathogens. It is even possible that the *stx1e* genes are an adaptation to the human or animal intestinal environment. Of course, the ability to detect Stx1e at low concentrations would help determine the prevalence of Stx-producing *E. cloacae* in the environment, agriculture, and ourselves.

The probability that the *stx1e* operon is carried upon an active phage and its similarity to other Stx1 subtypes provide clues to the origin of the *stx1e* operon. The *stx1e* operon has the greatest similarity to *stx1c*, but it has many unique polymorphisms in common with both *stx1d* and *stx/stx1a/stx1c*, although it is by far the most divergent Stx1 subtype (see Table S2 in the supplemental material). Most likely, *stx1e* diverged from *stx/stx1a/stx1c* alongside *stx1d* briefly and then evolved separately for a time (see Fig. S7 in the supplemental material). A possible mechanism for the considerable divergence of *stx1e* from the other *stx1* subtypes would be a change of the primary host. In this case, after the ancestral *stx1*-carrying phage adapted to stably infect *E. cloacae*, it evolved to *stx1e. E. cloacae* is common in the same environments as *E. coli* (part of the gut microbiota of humans and animals, as well as the areas where these animals live) (25), so it is likely that *E. cloacae* was exposed at some point to an *stx*-carrying phage. Since Stx1e is difficult to detect and not tested for, it is unknown how common Stx1e-expressing *E. cloacae* is. Therefore, it is difficult to predict whether the *stx1e* gene originated in ruminants or humans. However, if it can be proven that Stx1e gives *E. cloacae* a selective advantage against bacterial predators (such as *Tetrahymena*), it is more likely that Stx1e evolved in the environment or the intestines of ruminants. When more strains of Stx1e-expressing *E. cloacae* or *E. coli* surface, the question of Stx1e’s origin can be reevaluated.

10.1128/mSphere.00099-15.7 Figure S7  Phylogeny of Stx1 subtypes and Stx from *Shigella* spp. A phylogeny of Stx1 subtypes was constructed with Clustal 2.1 and the *stx1* operons (A and B subunits). Download Figure S7, PPTX file, 0.04 MB. Copyright © 2016 Skinner et al. 2016 Skinner et al. This content is distributed under the terms of the Creative Commons Attribution 4.0 International license.

10.1128/mSphere.00099-15.9 Table S2  Percent identity matrix for Stx1 subtypes and Stx. The *stx* operons were analyzed with Clustal 2.1. Download Table S2, PPTX file, 0.1 MB. Copyright © 2016 Skinner et al. 2016 Skinner et al. This content is distributed under the terms of the Creative Commons Attribution 4.0 International license.

The putative Stx1e phage itself is worthy of intense study. Atop the list of questions concerning the Stx1e phage is whether it has adapted to *E. cloacae* exclusively or if it is capable of infecting and disseminating the *stx1e* genes to other bacterial hosts. If the Stx1e phage is capable of infecting and propagating within *E. coli*, it is only a matter of time before STEC adds Stx1e to its repertoire of exotoxins. It may have happened already, since Stx1e is not routinely tested for and is difficult to detect by current methodologies. Similarly, it is possible that this phage originated in *E. coli* and is still present and undetected in some isolates, subsequently dispersing to *E. cloacae* as well.

An emerging Stx-producing *E. cloacae* pathogen and the new immunologically distinct Stx1e represent daunting challenges for health care, diagnostics, and food safety. However, these new antibodies and associated assays should prove capable of meeting these new challenges. The Stx1e-detecting assays developed in this study can be easily adapted to commercial assay platforms and made available for clinical diagnostic use. The availability of assays for Stx1e will be useful for determining the abundance of Stx1e-producing pathogens and addressing problems that these pathogens may cause.

## MATERIALS AND METHODS

### Strains used in this study.

The strains used in this study are described in Table 2.

**TABLE 2:** Strains used in this study

Strain	Genus and species	Biomolecule expressed	Origin	Reference
M12X01451	*E. cloacae*	Stx1e	Clinical	17
ATCC 13047	*E. cloacae*	No Stx	Clinical	
Stx1e (E167Q)	*E. coli*	Stx1e (E167Q)	Laboratory	This study
Stx1a (E167Q)	*E. coli*	Stx1a (E167Q)	Laboratory	27
Stx1c (E167Q)	*E. coli*	Stx1c (E167Q)	Laboratory	NA^a^
Stx1d (E167Q)	*E. coli*	Stx1d (E167Q)	Laboratory	NA
RM10638	*E. coli*	Stx2a	Cow (2009)	29
ATCC 25922	*E. coli*	No Stx	Clinical	

a NA, not available.

### Cloning, expression, and purification of Stx1e (E167Q) toxoid.

To generate a nontoxic immunogen, the E167Q point mutation was introduced into the A subunit of Stx1e by mutagenic PCR (26). The *stx1e* (E167Q) operon (including the A and B subunits of Stx1e) was constructed by mutagenic PCR (see Table S1 in the supplemental material) with gDNA prepared from strain M12X01451 of *E. cloacae* and incorporated into the pQE-T7-2 vector by methods previously described (26). This plasmid was transformed into BL21(DE3)pLysS competent cells, which were grown for 24 h in LB with 50 µg/ml kanamycin at 30°C and 150 rpm and then diluted 50-fold into 500 ml of LB plus 50 µg/ml kanamycin. They were grown for 4 h at 30°C and then induced with 1 mM isopropyl-β-d-thiogalactopyranoside (Sigma-Aldrich) overnight at 20°C at 150 rpm. Cells were centrifuged (5,500 × *g*, 15 min, 4°C), resuspended in PBS (50 mM Na_2_PO_4_, 150 mM NaCl, pH 7.4), and sonicated (40% amplitude, six 10-s pulses). Cell debris was centrifuged and discarded (5,000 × *g*, 15 min, 4°C), and then MnCl_2_ was added to the lysate at a final concentration of 50 mM. After the lysate was stirred at room temperature (RT, 22°C) for 10 min and centrifuged (5,500 × *g*, 30 min, 4°C), NH_4_SO_4_ was added to 70% saturation and protein was allowed to precipitate for 30 min during stirring on ice. Precipitated protein was centrifuged (5,500 × *g*, 30 min, 4°C) and resuspended in PBS. This was buffer changed to 20 mM Tris-HCl, pH 8.0, with a Zeba desalting column (Fisher Scientific). Anion-exchange chromatography was performed with an Akta fast protein liquid chromatograph and a HITRAP Q-HP column (GE Healthcare), and Stx1e (E167Q) was eluted with 20 mM Tris-HCl, pH 8.0, plus 1 M NaCl. Positive fractions were buffer exchanged to 50 mM NaPO_4_ plus 1 M NH_4_SO_4_, and hydrophobic interaction chromatography was performed with a HITRAP phenyl-HP column. Stx1e (E167Q) was eluted with 50 mM NaPO_4_ without NH_4_SO_4_. Positive fractions were concentrated with an Amicon Centricon (Fisher Scientific) and then subjected to gel filtration with a Sephadex 100 HiPrep column with PBS. Other Stx toxoids (Stx1a, Stx1c, Stx1d, and Stx2a), all with the E167Q mutation, were subjected to affinity purification (27).

### Expression and purification of native Stx1e toxin.

Stx1e-expressing strain *E. cloacae* M12X01451 was grown overnight at 37°C and 150 rpm in LB and then diluted 1/50 in LB with 50 ng/ml mitomycin C and grown for an additional 24 h. A 500-ml volume of this culture was centrifuged (5,500 × *g*, 15 min, 4°C), and the cell pellet was frozen (−20°C) overnight. The pellet was then thawed and resuspended in PBS, and lysozyme was added to 1 mg/ml (20 ml total). The suspension was then incubated on ice for 30 min and sonicated. The resulting lysate was then filter sterilized (0.2 µm), treated with MnCl_2_, precipitated with NH_4_SO_4_, anion exchanged, and subjected to hydrophobic interaction chromatography as with the Stx1e toxoid [see cloning, expression, and purification of Stx1e (E167Q) toxoid above]. Positive fractions from hydrophobic interaction chromatography were then purified with a MAb Stx1e-2 affinity column. The MAb Stx1e-2 affinity column was prepared with a MicroLink Protein Coupling kit (Pierce) according to the kit protocol. All handling steps were conducted in a biosafety level 2 cabinet.

### Immunization and hybridoma preparation, fusion, and screening.

SP2/0 myeloma cells and splenocytes were grown and prepared as previously described (6). Mice received a total of three intraperitoneal injections of 5 µg of Stx1e (E167Q) in the Sigma adjuvant system (Sigma-Aldrich) at 2-week intervals. Two weeks after the third injection, mice were boosted with 1 µg/mouse Stx1e (E167Q) in sterile PBS. Four days later, mice were sacrificed by rapid cervical dislocation, their spleens were excised, and splenocytes were harvested as previously described (6). Hybridomas were fused as previously described (6). Briefly, fusions of SP2/0 myeloma cells and splenocytes were achieved in accordance with a polyethylene glycol-based protocol. Clonal hybridoma lines were then obtained with three to five rounds of cloning by limited dilution, regrowth, and screening. Screening was conducted by direct ELISA.

### ELISAs.

Hybridoma screening ELISAs were conducted as previously described, with 100 ng/ml Stx1e (E167Q) as the plate-binding antigen and blocking buffer containing 5% nonfat dry milk in PBST (PBS with 0.5% Tween 20) (6). Sandwich ELISAs were also performed as previously described (6), with 5 µg/ml capture antibody, 1 µg/ml detection antibody, and blocking buffer containing 3% bovine serum albumin (BSA) in PBST. Antibodies used for detection were either an unconjugated PAb or a biotinylated MAb. A compatible HRP conjugate (streptavidin-HRP or goat anti-rabbit–HRP) at 0.2 µg/ml in BSA-PBST was then added to the ELISA well for a 1-h incubation. ELISAs were developed with SuperSignal West Pico chemiluminescent substrate (Pierce). All of the ELISAs presented in the figures shown here were performed at least three times, and a representative ELISA is shown. Human serum was purchased from Innovative Research, Inc.

### Western immunoblot assays.

Western immunoblot assays were conducted as previously described (6). Pure toxin/toxoid and cell-free medium samples were incubated at 72°C for 10 min in 1× NuPage lithium dodecyl sulfate (LDS) loading buffer and then run on a 4 to 12% NuPAGE Novex Bis-Tris minigel (Invitrogen). Cell lysate and cell-free medium samples were generated by centrifuging 100 µl of culture, removing the medium to a separate tube, adding LDS buffer to each sample (25 µl of 4× LDS for the medium sample, 125 µl of 1× LDS for the cell pellet), and then heating it at 95°C for 10 min at 1,400 rpm. Five microliters of each sample was then loaded onto a gel. The proteins were then transferred to a polyvinylidene difluoride membrane (pore size, 0.45 µm; Amersham Hybond-P), blocked with 2% ECL Prime blocking agent (GE Healthcare) in PBST, and washed three times with PBST. MAbs were diluted to 1 µg/ml in blocking buffer and incubated with the blot assays for 1 h at RT, and then the blot assays were washed three times in PBST. GAM-HRP antibody (Promega) at a 1/20,000 dilution was incubated on the blot for 1 h at RT. The blots were washed four more times with PBST (5 min each time) and developed with Lumigen TMA-6 (Lumigen) substrate. The blot assays were visualized with a 5-min exposure with a FluorChem HD2 (Alpha Innotech). All Western blot assays were conducted three times, and a representative blot is shown.

### Vero cell cytotoxicity and antibody neutralization assays.

Vero cells were prepared and grown as previously described (28). Before the cytotoxicity assays were conducted, the cells were trypsinized, diluted to 10^5^/ml, dispensed into 96-well cell culture-treated plates, and then incubated for 24 h. Filter-sterilized mitomycin C-induced M12X01451 culture medium or pure Stx1e toxin was diluted in fresh Vero medium (0.1 µl of medium/well for cell-free medium). Purified Stx1a from a crude preparation (Toxin Technologies, subjected to affinity purification) (27) was used as a control. MAbs were preincubated at 10 µg/ml with toxin-containing medium for 1 h at RT before the mixture was added to the Vero cells. The medium in the Vero cell assay plate was then removed and replaced with the Stx1e and/or MAb-containing mixture (100 µl/well). Twenty-four hours after treatment, the Vero cells were lysed with 100 µl/well CellTitre-Glo reagent (Promega) diluted 1:3 in PBS with 3 min of shaking. Luminescence was measured with a Victor II plate reader. All Vero cell toxicity assays were conducted three times with similar results. Photographs were obtained from replicate wells with a Cytation 3 module at ×200 magnification.

### PCR and Benzonase treatment.

With the exception of those used to construct the Stx1e (E167Q) toxoid, all of the PCR samples contained filter-sterilized (0.2 µm) medium from M12X01451 or *E. cloacae* (ATCC 13047). Benzonase treatment consisted of adding 2.5 U of Benzonase to 50 µl of filtered medium and incubating it at 37°C for 15 min. Samples were then concentrated with a 100-kDa Amicon filter and washed. *stx1e* colony PCRs were conducted with the GoTaq Green master mix (Promega) and primers Stx1e-spec-F1 and Stx1e-spec-R1 (see Table S1 in the supplemental material). *wrbA* PCRs were conducted with GoTaq Green and primers wrbA-F1 and wrbA-R1 (see Table S1 in the supplemental material). gDNA (a total of 6 ng per sample from strain M12X01451) was used to spike control samples. A 10-min incubation step at 95°C was included to denature all viral or bacterial DNA before PCR. This was followed by 30 cycles of 1 min each of denaturation at 95°C, annealing at 55°C, and extension at 72°C. PCRs were analyzed by gel electrophoresis with 0.8% agarose in Tris-acetate-EDTA and visualized under UV light with GelRed (Biotium).

### Antibody affinity measurements.

The affinity of the MAbs for Stx1e (E167Q) was measured with an Octet QK^e^ system (*forté*BIO, Menlo Park, CA) as previously described (6). Biotinylated MAbs were bound to streptavidin biosensors at 10 µg/ml in PBS. Stx1e (E167Q) was then incubated with the sensors at different concentrations (142, 71, 36, and 18 nM) and then allowed to dissociate in PBS. Dissociation constants (*K_D_* values) were calculated with the Octet QK^e^ software (Data acquisition 7.0).

### Receptor binding assay.

Gb3/Gb4 binding assays were conducted as previously described (28). Briefly, fixed *E. coli* cells expressing Gb3-LPS, Gb4-LPS, or a control were diluted to an optical density at 600 nm of 0.05 in carbonate buffer (0.1 M NaCO_3_, pH 9.6), and 100 µl was bound to the wells of an ELISA plate by incubation at 50°C until all of the liquid had evaporated. Wells were then blocked with 5% milk–PBST for 1 h. Stx1e was then added at the indicated concentrations, and the mixture was incubated for 1 h at RT. MAb Stx1e-2 at a 1/1,000 dilution in blocking buffer was then added, and the mixture was incubated for 1 h. Goat anti-mouse IgG-HRP at 1/5,000 was then added, and the mixture was incubated for 1 h. The signal was detected with SuperSignal West Pico chemiluminescent substrate (Thermo Scientific), and plates were read on a Victor II plate reader (PerkinElmer). Wells were washed six times with PBST between incubations.

## References

[1] Franklin AB, Vercauteren KC, Maguire H, Cichon MK, Fischer JW, Lavelle MJ, Powell A, Root JJ, Scallan E 2013 Wild ungulates as disseminators of Shiga toxin-producing *Escherichia coli* in urban areas. PLoS One 8:e81512. doi:10.1371/journal.pone.0081512. 24349083 PMC3859483

[2] Majowicz SE, Scallan E, Jones-Bitton A, Sargeant JM, Stapleton J, Angulo FJ, Yeung DH, Kirk MD 2014 Global incidence of human Shiga toxin-producing Escherichia coli infections and deaths: a systematic review and knowledge synthesis. Foodborne Pathog Dis 11:447–455. doi:10.1089/fpd.2013.1704. 24750096 PMC4607253

[3] Zschöck M, Hamann HP, Kloppert B, Wolter W 2000 Shiga-toxin-producing Escherichia coli in faeces of healthy dairy cows, sheep and goats: prevalence and virulence properties. Lett Appl Microbiol 31:203–208. doi:10.1046/j.1365-2672.2000.00789.x. 10972729

[4] Rüssmann H, Schmidt H, Heesemann J, Caprioli A, Karch H 1994 Variants of Shiga-like toxin II constitute a major toxin component in Escherichia coli O157 strains from patients with haemolytic uraemic syndrome. J Med Microbiol 40:338–343. doi:10.1099/00222615-40-5-338. 7909850

[5] Friedrich AW, Bielaszewska M, Zhang WL, Pulz M, Kuczius T, Ammon A, Karch H 2002 Escherichia coli harboring Shiga toxin 2 gene variants: frequency and association with clinical symptoms. J Infect Dis 185:74–84. doi:10.1086/338115. 11756984

[6] Skinner C, Patfield S, Stanker L, He X 2013 Development of monoclonal antibodies and immunoassays for sensitive and specific detection of Shiga toxin stx2f. PLoS One 8:e76563. doi:10.1371/journal.pone.0076563. 24069462 PMC3775747

[7] Karve SS, Weiss AA 2014 Glycolipid binding preferences of Shiga toxin variants. PLoS One 9:e101173. doi:10.1371/journal.pone.0101173. 24983355 PMC4077739

[8] Fuller CA, Pellino CA, Flagler MJ, Strasser JE, Weiss AA 2011 Shiga toxin subtypes display dramatic differences in potency. Infect Immun 79:1329–1337. doi:10.1128/IAI.01182-10. 21199911 PMC3067513

[9] Willford J, Mills K, Goodridge LD 2009 Evaluation of three commercially available enzyme-linked immunosorbent assay kits for detection of Shiga toxin. J Food Prot 72:741–747. 19435221 10.4315/0362-028x-72.4.741

[10] McDonough MA, Butterton JR 1999 Spontaneous tandem amplification and deletion of the Shiga toxin operon in Shigella dysenteriae 1. Mol Microbiol 34:1058–1069. doi:10.1046/j.1365-2958.1999.01669.x. 10594830

[11] Schmidt H, Montag M, Bockemühl J, Heesemann J, Karch H 1993 Shiga-like toxin II-related cytotoxins in *Citrobacter freundii* strains from humans and beef samples. Infect Immun 61:534–543. 8423084 10.1128/iai.61.2.534-543.1993 PMC302761

[12] Tschape H, Prager R, Streckel W, Fruth A, Tietze E, Böhme G 1995 Verotoxinogenic Citrobacter freundii associated with severe gastroenteritis and cases of haemolytic uraemic syndrome in a nursery school: green butter as the infection source. Epidemiol Infect 114:441–450. doi:10.1017/S0950268800052158. 7781732 PMC2271295

[13] Paton AW, Paton JC 1996 *Enterobacter cloacae* producing a Shiga-like toxin II-related cytotoxin associated with a case of hemolytic-uremic syndrome. J Clin Microbiol 34:463–465. 8789041 10.1128/jcm.34.2.463-465.1996 PMC228823

[14] Grotiuz G, Sirok A, Gadea P, Varela G, Schelotto F 2006 Shiga toxin 2-producing *Acinetobacter haemolyticus* associated with a case of bloody diarrhea. J Clin Microbiol 44:3838–3841. doi:10.1128/JCM.00407-06. 17021124 PMC1594762

[15] Alperi A, Figueras MJ 2010 Human isolates of Aeromonas possess Shiga toxin genes (stx1 and stx2) highly similar to the most virulent gene variants of Escherichia coli. Clin Microbiol Infect 16:1563–1567. doi:10.1111/j.1469-0691.2010.03203.x. 20219084

[16] Casas V, Sobrepeña G, Rodriguez-Mueller B, Ahtye J, Maloy SR 2011 Bacteriophage-encoded Shiga toxin gene in atypical bacterial host. Gut Pathog 3:10. doi:10.1186/1757-4749-3-10. 21733190 PMC3146822

[17] Probert WS, McQuaid C, Schrader K 2014 Isolation and identification of an *Enterobacter cloacae* strain producing a novel subtype of Shiga toxin type 1. J Clin Microbiol 52:2346–2351. doi:10.1128/JCM.00338-14. 24759708 PMC4097712

[18] Scheutz F, Teel LD, Beutin L, Piérard D, Buvens G, Karch H, Mellmann A, Caprioli A, Tozzoli R, Morabito S, Strockbine NA, Melton-Celsa AR, Sanchez M, Persson S, O’Brien AD 2012 Multicenter evaluation of a sequence-based protocol for subtyping Shiga toxins and standardizing Stx nomenclature. J Clin Microbiol 50:2951–2963. doi:10.1128/JCM.00860-12. 22760050 PMC3421821

[19] Goswami K, Chen C, Xiaoli L, Eaton KA, Dudley EG 2015 Coculture of *Escherichia coli* O157:H7 with a nonpathogenic *E. coli* strain increases toxin production and virulence in a germfree mouse model. Infect Immun 83:4185–4193. doi:10.1128/IAI.00663-15. 26259815 PMC4598395

[20] Dubos RJ, Geiger JW 1946 Preparation and properties of Shiga toxin and toxoid. J Exp Med 84:143–156. doi:10.1084/jem.84.2.143. 20994379

[21] Calderwood SB, Mekalanos JJ 1987 Iron regulation of Shiga-like toxin expression in *Escherichia coli* is mediated by the *fur* locus. J Bacteriol 169:4759–4764. 3308853 10.1128/jb.169.10.4759-4764.1987 PMC213851

[22] Gallegos KM, Conrady DG, Karve SS, Gunasekera TS, Herr AB, Weiss AA 2012 Shiga toxin binding to glycolipids and glycans. PLoS One 7:e30368. doi:10.1371/journal.pone.0030368. 22348006 PMC3278406

[23] Paton AW, Morona R, Paton JC 2000 A new biological agent for treatment of Shiga toxigenic Escherichia coli infections and dysentery in humans. Nat Med 6:265–270. doi:10.1038/73111. 10700227

[24] Paton AW, Morona R, Paton JC 2001 Neutralization of Shiga toxins Stx1, Stx2c, and Stx2e by recombinant bacteria expressing mimics of globotriose and globotetraose. Infect Immun 69:1967–1970. doi:10.1128/IAI.69.3.1967-1970.2001. 11179385 PMC98114

[25] Kim SH, Wei CI 2007 Expression of AmpC beta-lactamase in Enterobacter cloacae isolated from retail ground beef, cattle farm and processing facilities. J Appl Microbiol 103:400–408. doi:10.1111/j.1365-2672.2006.03255.x. 17650200

[26] He X, McMahon S, Skinner C, Merrill P, Scotcher MC, Stanker LH 2013 Development and characterization of monoclonal antibodies against Shiga toxin 2 and their application for toxin detection in milk. J Immunol Methods 389:18–28. doi:10.1016/j.jim.2012.12.005. 23279946

[27] Skinner C, Patfield S, Stanker LH, Fratamico P, He X 2014 New high-affinity monoclonal antibodies against Shiga toxin 1 facilitate the detection of hybrid Stx1/Stx2 *in vivo*. PLoS One 9:e99854. doi:10.1371/journal.pone.0099854. 24914553 PMC4051773

[28] Skinner C, McMahon S, Rasooly R, Carter JM, He X 2013 Purification and characterization of Shiga toxin 2f, an immunologically unrelated subtype of Shiga toxin 2. PLoS One 8:e59760. doi:10.1371/journal.pone.0059760. 23555772 PMC3608586

[29] He X, Quiñones B, McMahon S, Mandrell RE 2012 A single-step purification and molecular characterization of functional Shiga toxin 2 variants from pathogenic *Escherichia coli*. Toxins 4:487–504. doi:10.3390/toxins4070487. 22852065 PMC3407889

