# Supplementary figures and images for: New Monoclonal Antibodies against a Novel Subtype of Shiga Toxin 1 Produced by Enterobacter cloacae and Their Use in Analysis of Human Serum
Source: mSphere. 2016 Feb 17;1(1):e00099-15. doi: 10.1128/mSphere.00099-15 (PMC4863616; doi:10.1128/mSphere.00099-15)

## Slide 1
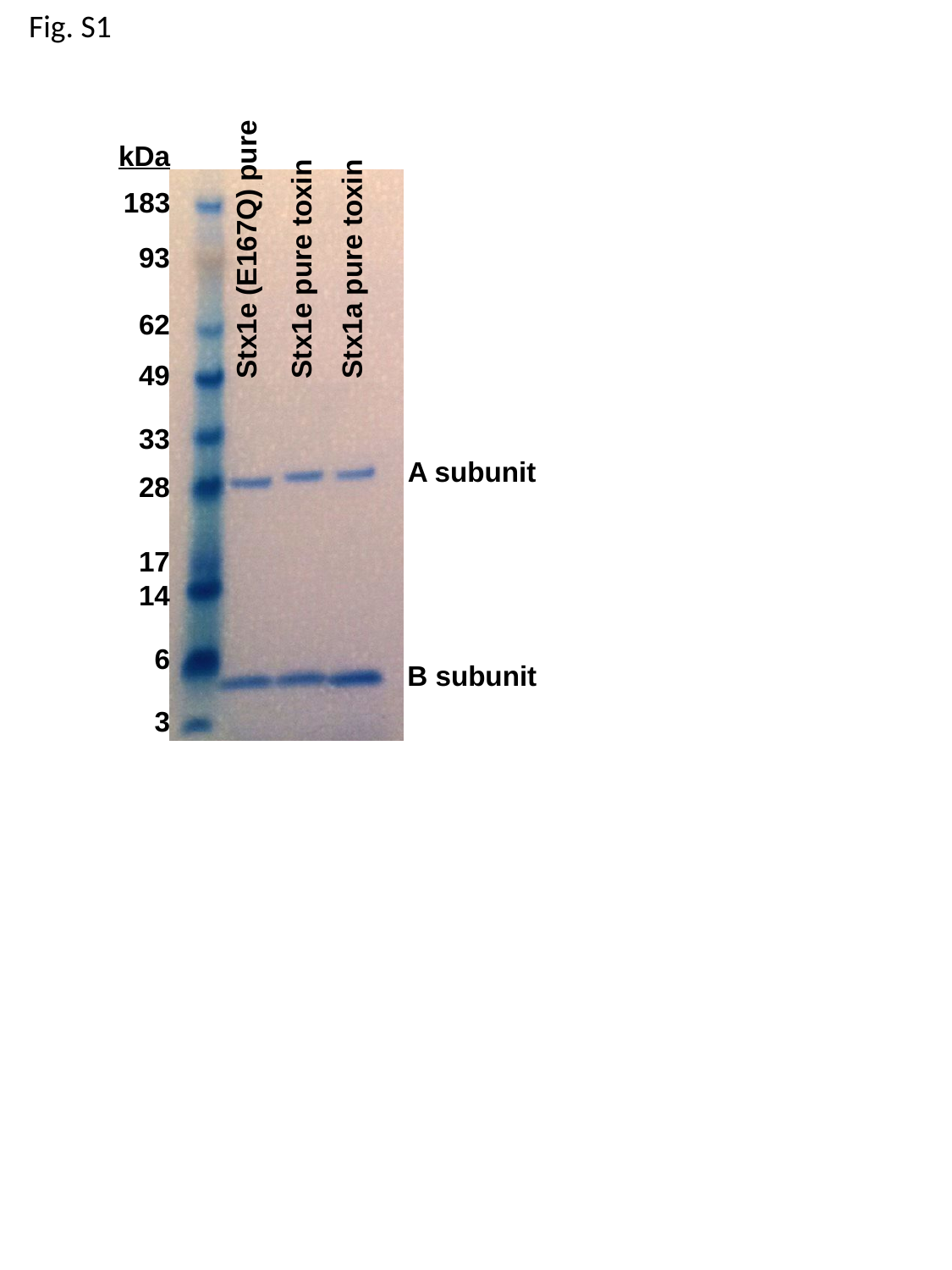

Fig. S1
kDa
183
93
62
49
33
28
17
14
6
3
Stx1e (E167Q) pure
Stx1e pure toxin
Stx1a pure toxin
A subunit
B subunit

Supplement: Figure S1 [file sph001162029sf1.pptx]

## Slide 1
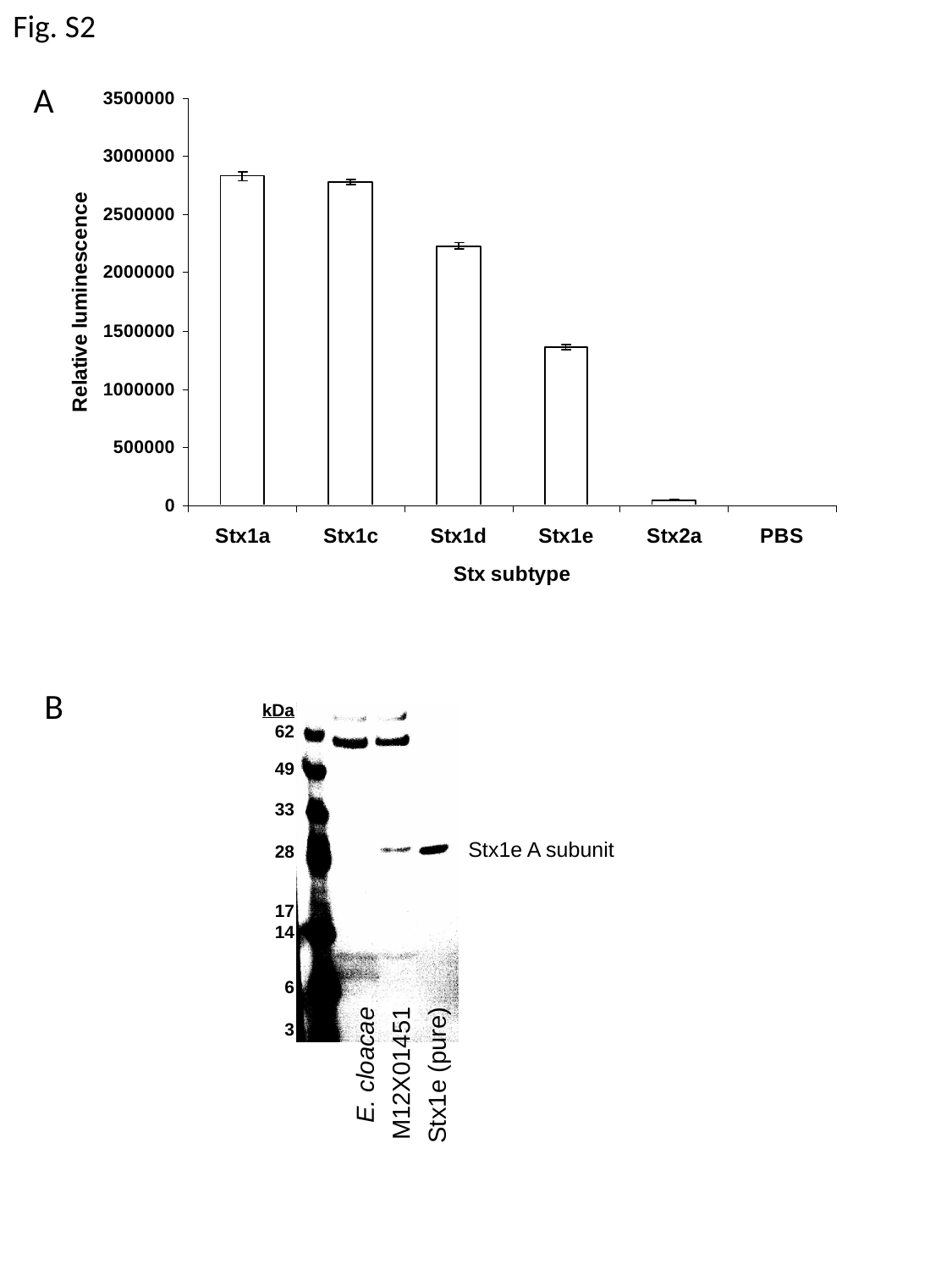

Fig. S2
A
B
kDa
62
49
33
28
17
14
6
3
Stx1e A subunit
E. cloacae
M12X01451
Stx1e (pure)

Supplement: Figure S2 [file sph001162029sf2.pptx]

## Slide 1
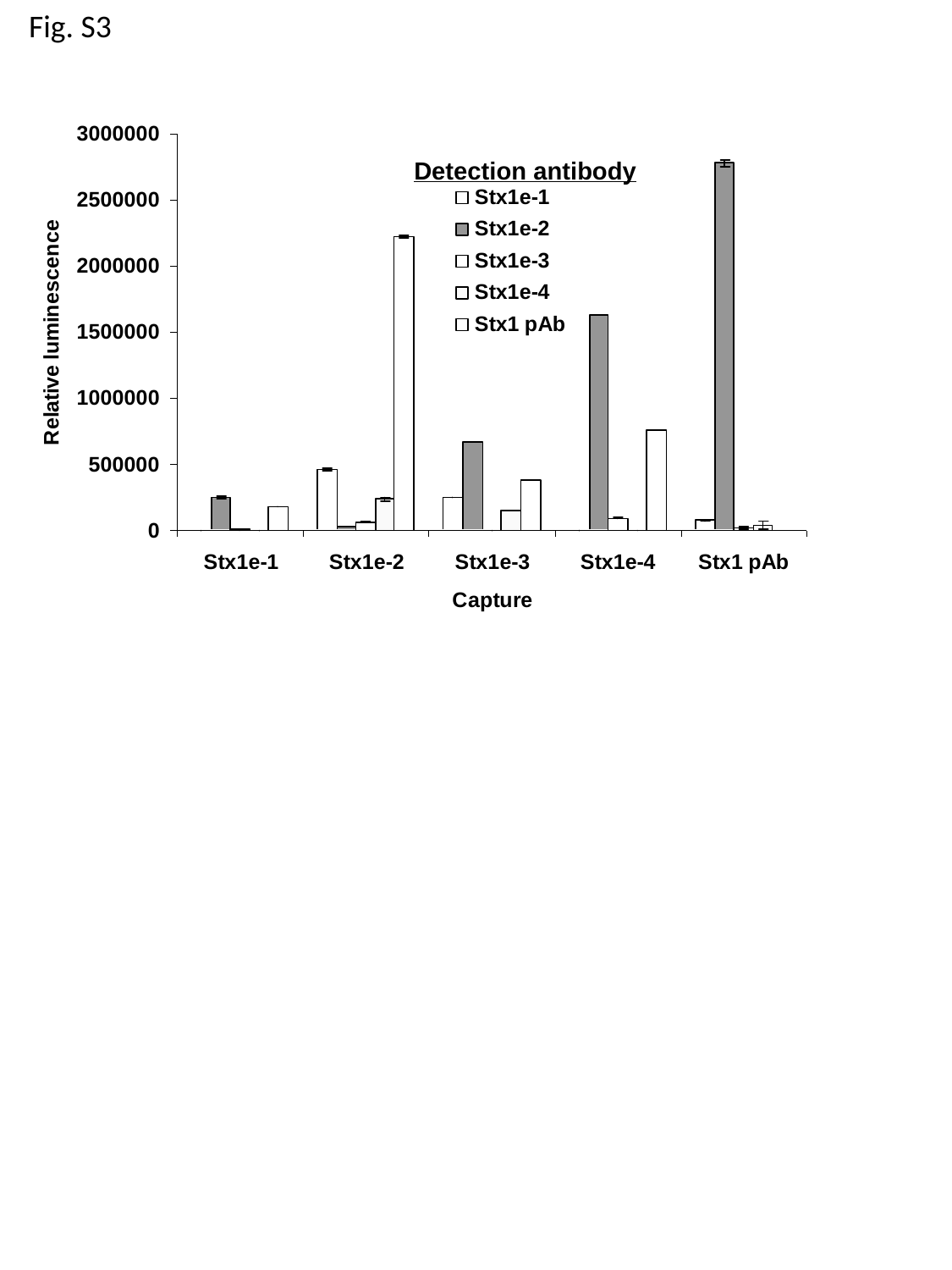

Fig. S3
Detection antibody

Supplement: Figure S3 [file sph001162029sf3.pptx]

## Slide 1
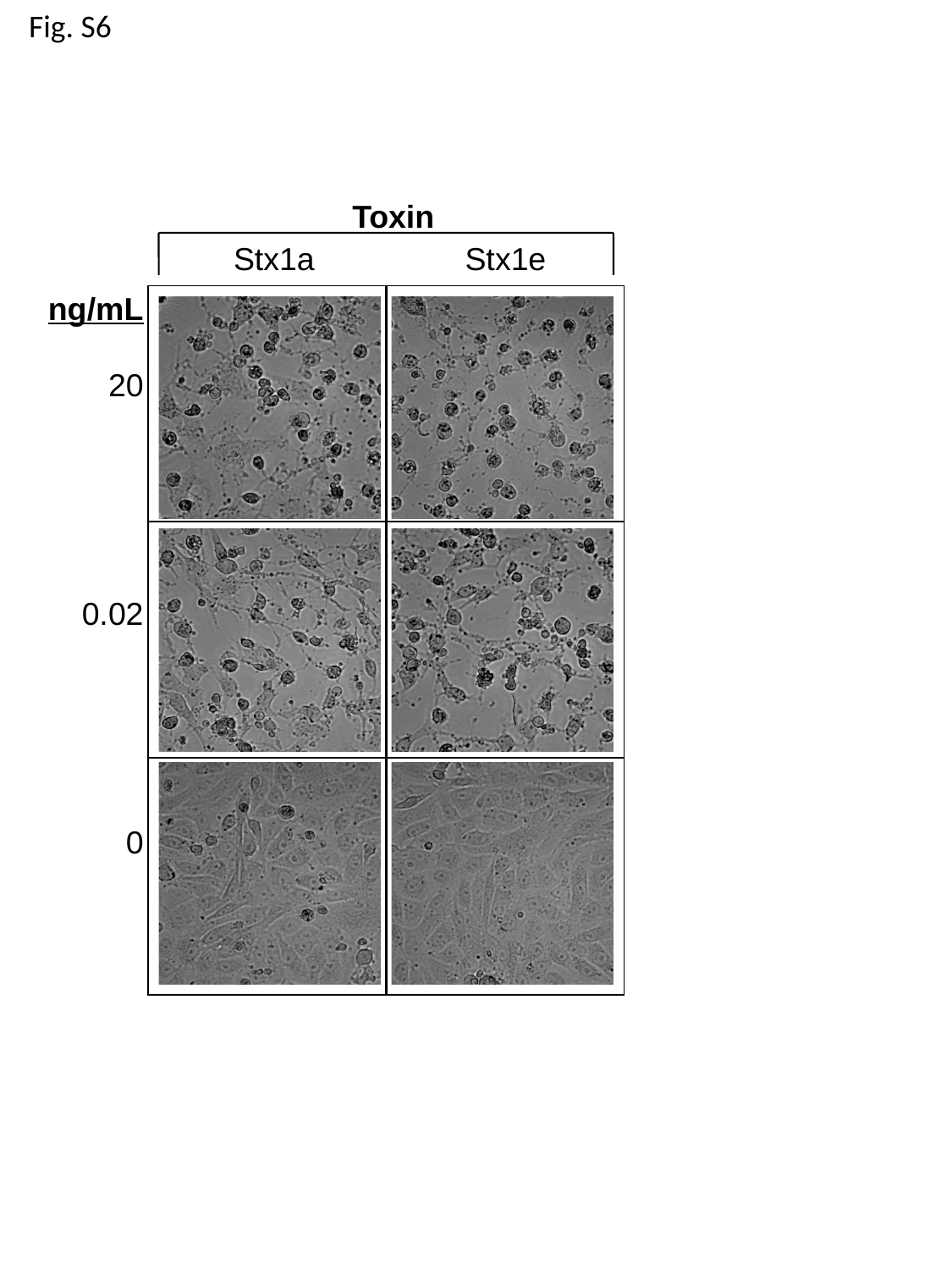

Fig. S6
Toxin
Stx1a Stx1e
ng/mL
20
0.02
0

Supplement: Figure S6 [file sph001162029sf6.pptx]

## Slide 1
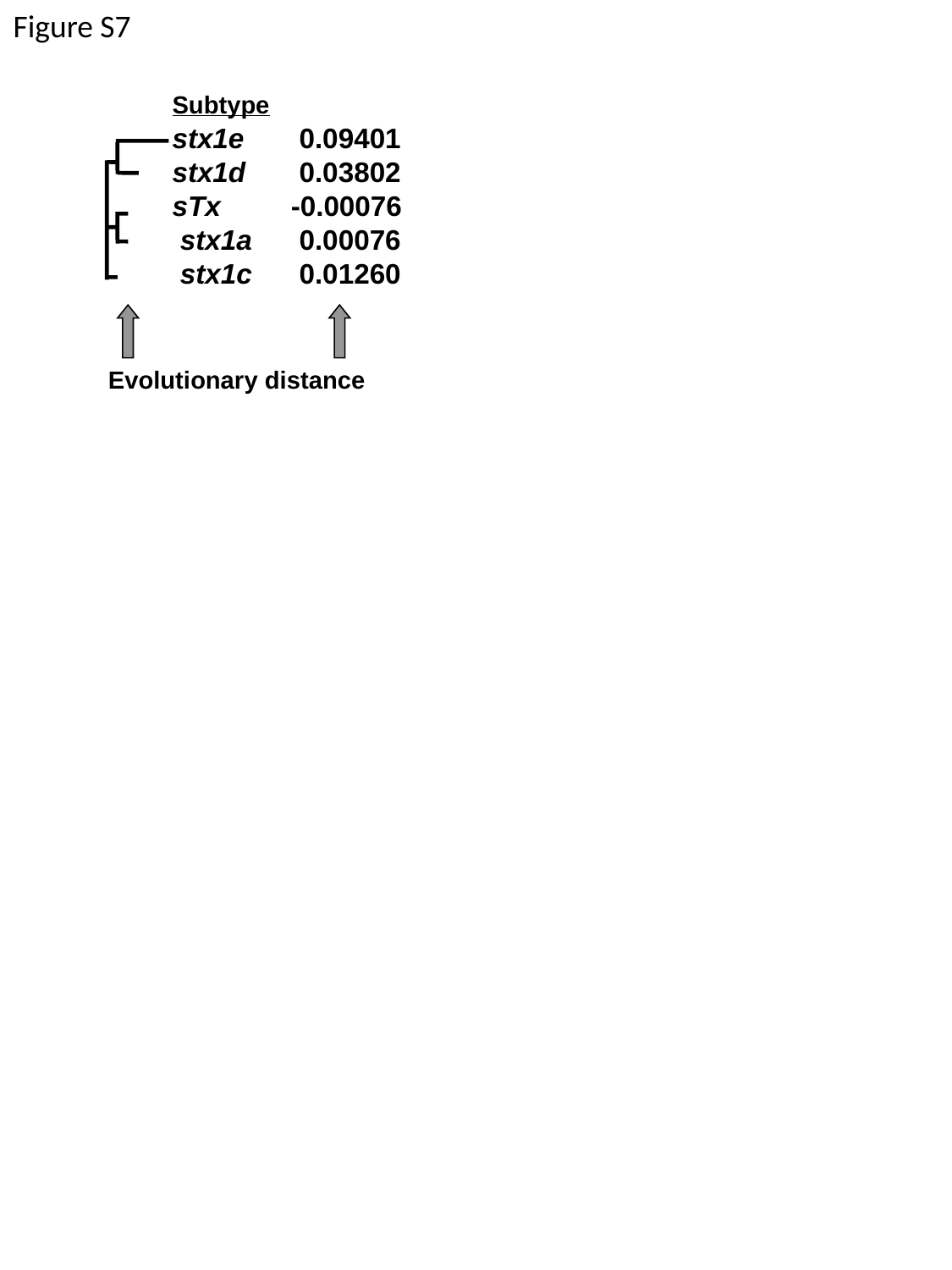

Figure S7
Subtype
stx1e	0.09401
stx1d	0.03802
sTx -0.00076
 stx1a	0.00076
 stx1c	0.01260
Evolutionary distance

Supplement: Figure S7 [file sph001162029sf7.pptx]

## Slide 1
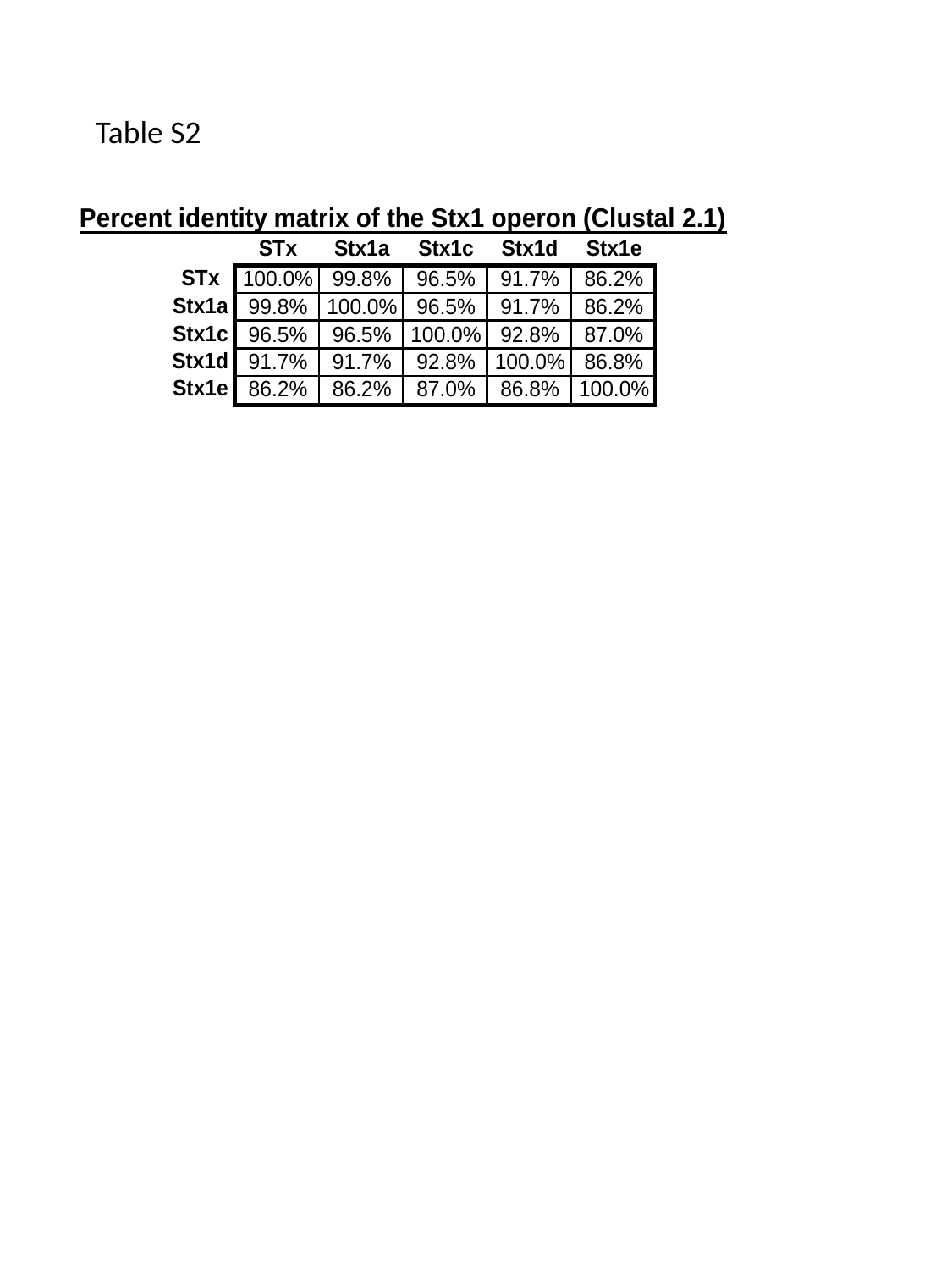

Table S2

Supplement: Table S2 [file sph001162029st9.pptx]
