# Supplementary material for: New Monoclonal Antibodies against a Novel Subtype of Shiga Toxin 1 Produced by Enterobacter cloacae and Their Use in Analysis of Human Serum
Source: mSphere. 2016 Feb 17;1(1):e00099-15. doi: 10.1128/mSphere.00099-15 (PMC4863616; doi:10.1128/mSphere.00099-15)
Supplement: Figure S4 [file sph001162029sf4.pptx]

## Slide 1
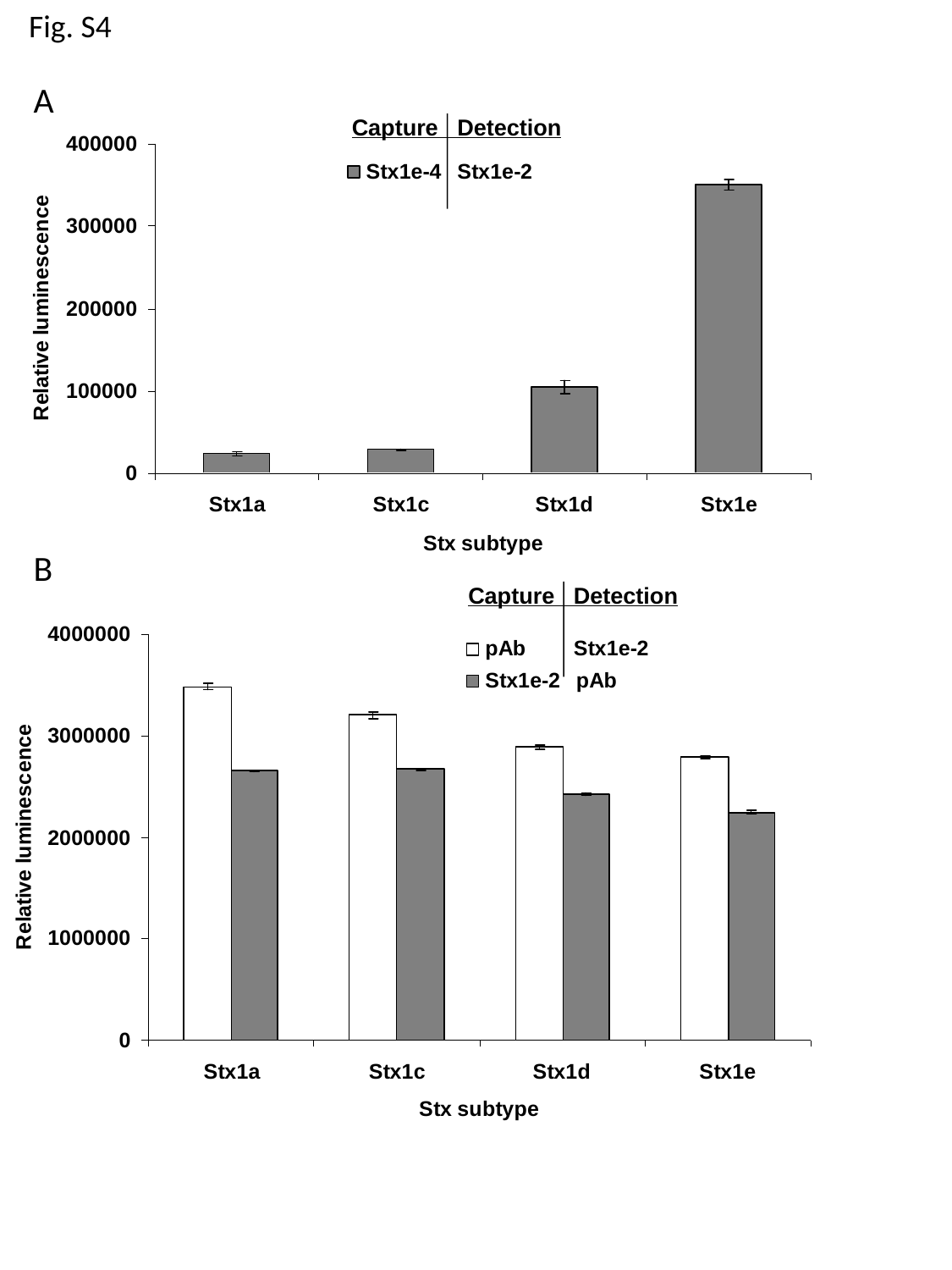

Fig. S4
A
Capture Detection
Capture Detection
Capture Detection
Capture Detection
B
Capture Detection
Capture Detection
