# Supplementary material for: New Monoclonal Antibodies against a Novel Subtype of Shiga Toxin 1 Produced by Enterobacter cloacae and Their Use in Analysis of Human Serum
Source: mSphere. 2016 Feb 17;1(1):e00099-15. doi: 10.1128/mSphere.00099-15 (PMC4863616; doi:10.1128/mSphere.00099-15)
Supplement: Figure S5 [file sph001162029sf5.pptx]

## Slide 1
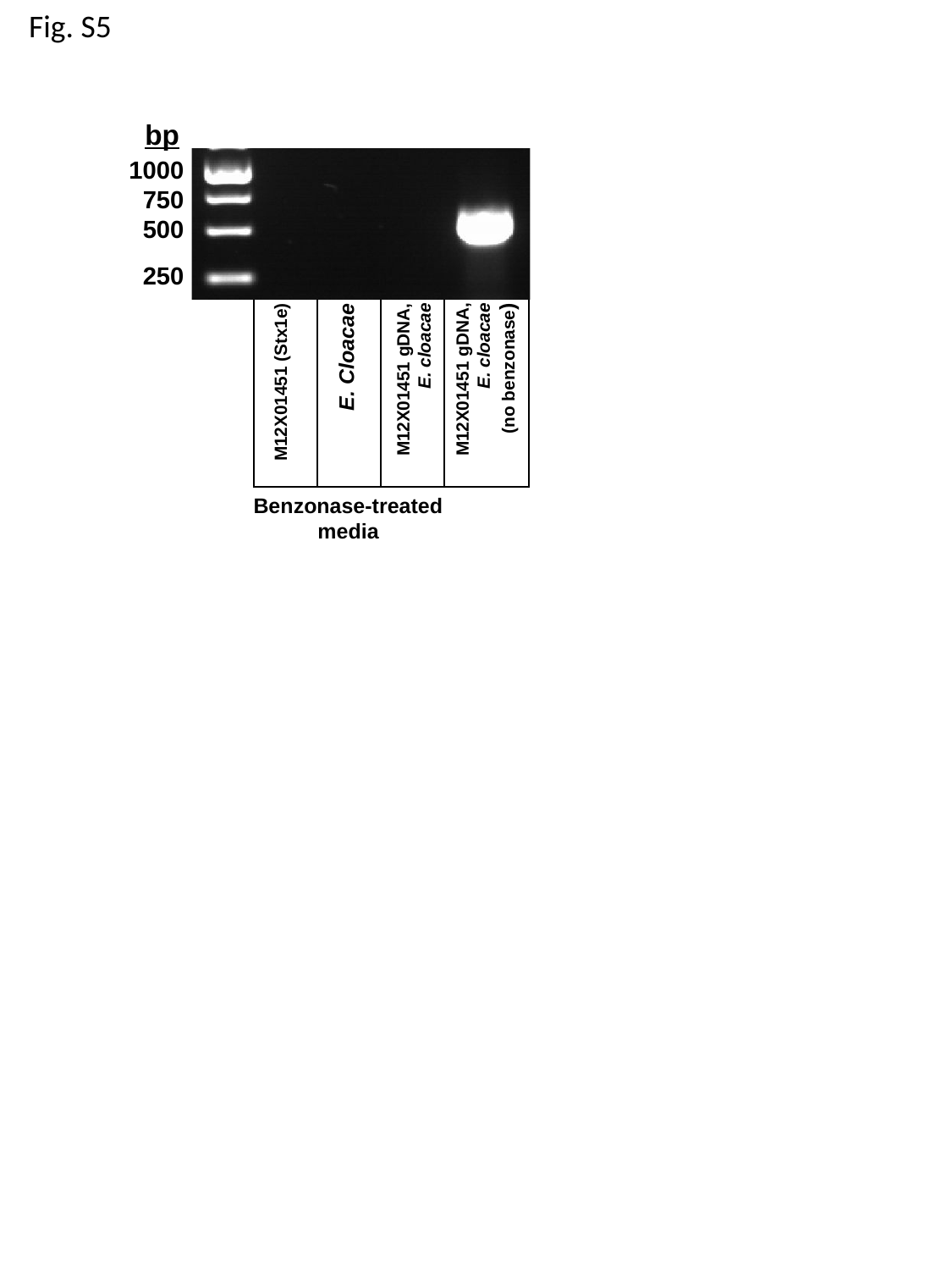

Fig. S5
bp
1000
750
500
250
M12X01451 (Stx1e)
E. Cloacae
M12X01451 gDNA,
E. cloacae
M12X01451 gDNA,
E. cloacae
(no benzonase)
Benzonase-treated
media
