# Supplementary material for: New Monoclonal Antibodies against a Novel Subtype of Shiga Toxin 1 Produced by Enterobacter cloacae and Their Use in Analysis of Human Serum
Source: mSphere. 2016 Feb 17;1(1):e00099-15. doi: 10.1128/mSphere.00099-15 (PMC4863616; doi:10.1128/mSphere.00099-15)
Supplement: Table S1 [file sph001162029st8.pptx]

## Slide 1
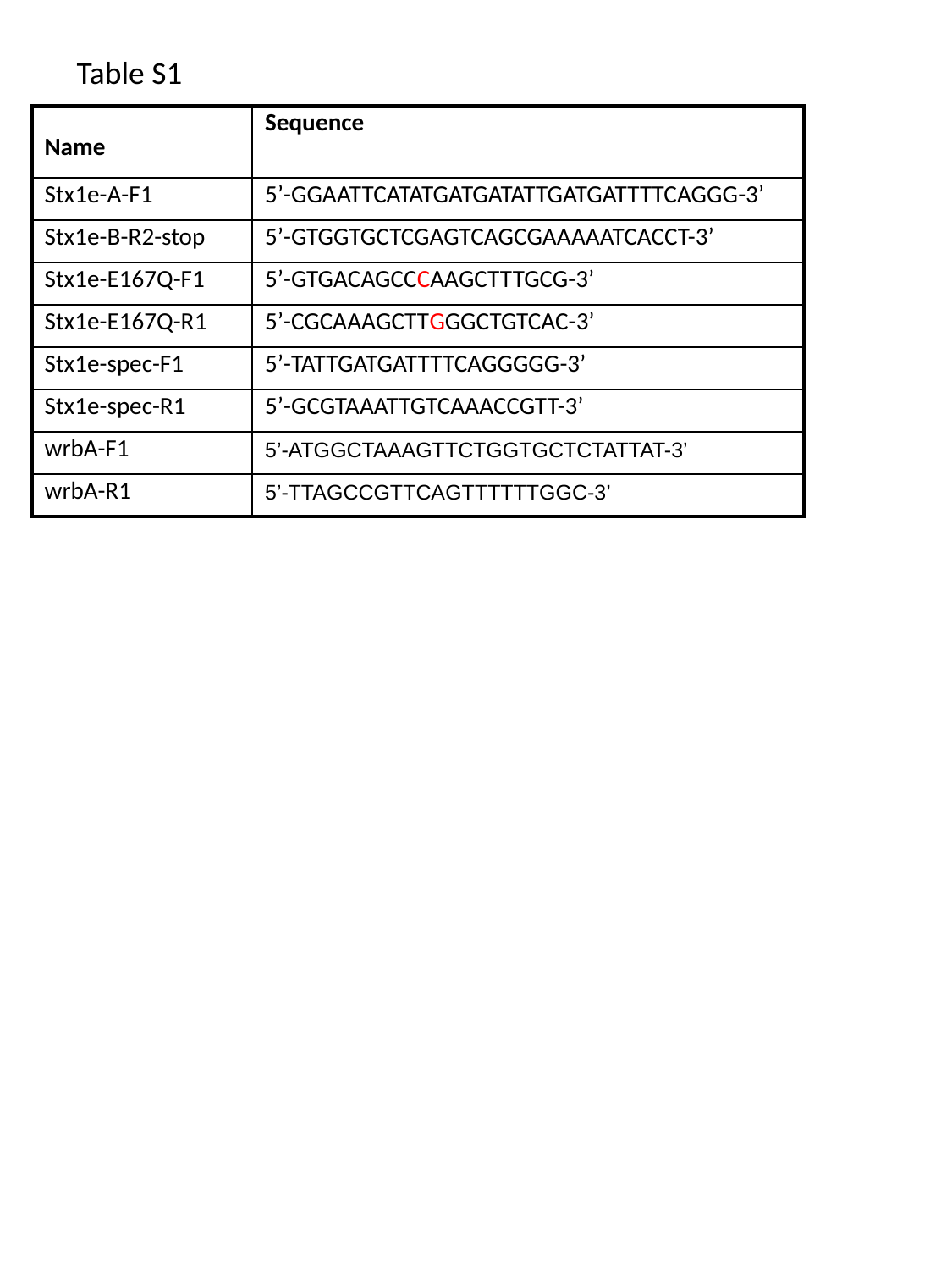

Table S1
| Name | Sequence |
| --- | --- |
| Stx1e-A-F1 | 5’-GGAATTCATATGATGATATTGATGATTTTCAGGG-3’ |
| Stx1e-B-R2-stop | 5’-GTGGTGCTCGAGTCAGCGAAAAATCACCT-3’ |
| Stx1e-E167Q-F1 | 5’-GTGACAGCCCAAGCTTTGCG-3’ |
| Stx1e-E167Q-R1 | 5’-CGCAAAGCTTGGGCTGTCAC-3’ |
| Stx1e-spec-F1 | 5’-TATTGATGATTTTCAGGGGG-3’ |
| Stx1e-spec-R1 | 5’-GCGTAAATTGTCAAACCGTT-3’ |
| wrbA-F1 | 5’-ATGGCTAAAGTTCTGGTGCTCTATTAT-3’ |
| wrbA-R1 | 5’-TTAGCCGTTCAGTTTTTTGGC-3’ |
